# Fluorescence Detection of Deoxyadenosine in *Cordyceps* spp. by Indicator Displacement Assay

**DOI:** 10.3390/molecules25092045

**Published:** 2020-04-28

**Authors:** Arinta Agnie Dewantari, Nattha Yongwattana, Panwajee Payongsri, Sawinee Seemakhan, Suparerk Borwornpinyo, Akio Ojida, Jirarut Wongkongkatep

**Affiliations:** 1 Department of Biotechnology, Faculty of Science, Mahidol University, Bangkok 10400, Thailand; arinta.agnie@gmail.com (A.A.D.); oun.nattha@gmail.com (N.Y.); panwajee.pay@mahidol.ac.th (P.P.); suparerk.bor@mahidol.ac.th (S.B.); 2 Excellent Center for Drug Discovery, Faculty of Science, Mahidol University, Bangkok 10400, Thailand; sawinee.ecdd@gmail.com; 3 Graduate School of Pharmaceutical Sciences, Kyushu University, 3-1-1 Maidashi, Higashi-ku, Fukuoka 812-8582, Japan; ojida@phar.kyushu-u.ac.jp

**Keywords:** cordycepin, fungi, indicator displacement assay, fluorescence, detection, cucurbit uril, acridine orange

## Abstract

A rapid, sensitive and reliable indicator displacement assay (IDA) for specific detection of 2′- and 3′-deoxyadenosine (2′-dAde and 3′-dAde), the latter is also known as cordycepin, was established. The formation of inclusion complex between protonated acridine orange (AOH^+^) and cucurbit[7]uril (CB7) resulted in the hypochromic shift of fluorescent emission from 530 nm to 512 nm. Addition of cordycepin to the highly fluorescent AOH^+^/CB7 complex resulted in a unique tripartite AOH^+^/CB7/dAde complex with diminished fluorescence, and such reduction in emission intensity serves as the basis for our novel sensing system. The detection limits were 11 and 82 μM for 2′- and 3′-deoxyadenosine, respectively. The proposed method also demonstrated high selectivity toward 2′- and 3′-deoxyadenosine, owing to the inability of other deoxynucleosides, nucleosides and nucleotides commonly found in *Cordyceps* spp. to displace the AOH^+^ from the AOH^+^/CB7 complex, which was confirmed by isothermal titration calorimetry (ITC), UV-Visible and proton nuclear magnetic resonance (^1^H-NMR) spectroscopy. Our method was successfully implemented in the analysis of cordycepin in commercially available *Ophiocordyceps* and *Cordyceps* supplements, providing a novel and effective tool for quality assessment of these precious fungi with several health benefits.

## 1. Introduction

Cordycepin, also commonly known as 3′-deoxyadenosine, has gained increasing attention in cancer therapy due to its anti-tumour properties. For instance, it was previously reported that administration of cordycepin to mice with oral cancer can prolong survival and slow down epithelial-mesenchymal transition [1]. In addition, cordycepin was found to trigger apoptosis, leading to reduced proliferation of human lung cancer cell line and gallbladder cancer cells [2,3]. The major sources of cordycepin are *Ophiocordyceps* and *Cordyceps* fungi, which have been used for a long time in folk oriental medicine to treat several symptoms including fatigue, respiratory diseases, renal dysfunction, arrhythmias and other heart diseases [4,5]. Several studies have also attested to the pharmacological benefits of *Ophiocordyceps* and *Cordyceps* extracts, including antibacterial, anticancer, antifungal, anti-inflammatory, and antioxidant properties [6,7,8].

Capillary electrophoresis, HPLC and LC-MS/MS have been developed for the determination of cordycepin and other nucleosides in *Ophiocordyceps* and *Cordyceps*, but they possess drawbacks in terms of separation efficiency for polar compounds [9,10,11,12,13] and produce lots of chemical waste. Recently, a hydrophilic interaction chromatography (HILIC) is coupled with an electrospray ionization tandem mass spectrometry (ESI-MS/MS) was reported for detection of cordycepin [14]. This HILIC ESI-MS/MS method showed high sensitivity, dynamic range, repeatability and recovery relative to routine HPLC. Compared with chromatographic methods, however, fluorescence assays generally allow for superior sensitivity, have rapid and simplified procedures, and generate less chemical wastes.

**Figure 1:**
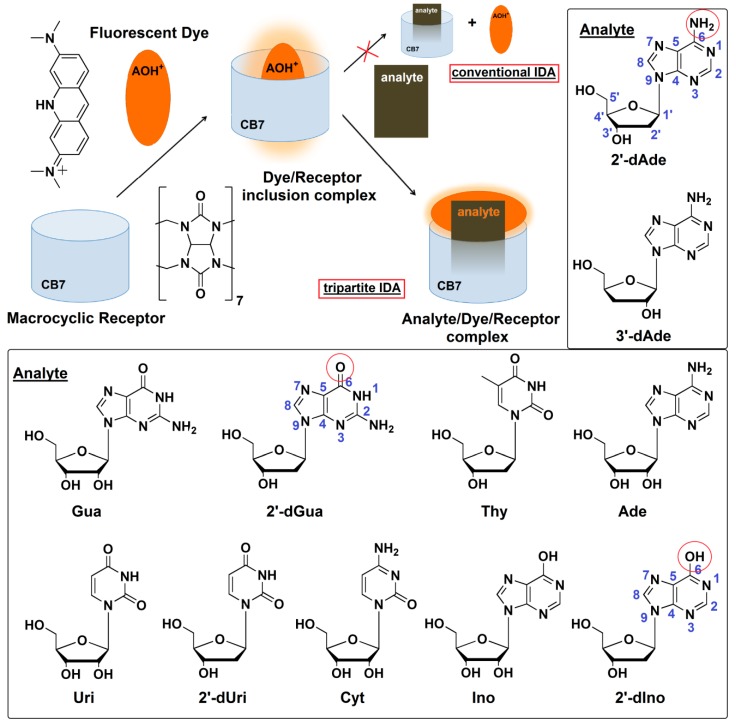
Proposed mechanism of a newly developed tripartite IDA strategy using protonated acridine orange (AOH^+^) and cucurbit[7]uril (CB7) based on the transformation from inclusion to Exo complex of AOH^+^ for detection of bioactive nucleosides found in *Cordyceps* spp. Ade: adenosine, 2′-dAde: 2′-deoxyadenosine, 3′-dAde: 3′-deoxyadenosine or cordycepin, Ino: inosine, 2′-dIno: 2′-deoxyinosine, Gua: guanosine, 2′-dGua: 2′-deoxyguanosine, Uri: uridine, 2′-dUri: 2′-deoxyuridine, Cyt: cytidine, Thy: thymidine.

Indicator displacement assay (IDA) using fluorescent dye is a supramolecular analytical method which employs the concept of “self-molecular recognition” through the supramolecular interactions such as hydrogen bonds, salt bridges, π-π stacking, van der Waals forces and hydrophobic interactions [15]. In a conventional IDA experiment, an indicator is first bound to a receptor, creating the sensing unit. An analyte was introduced subsequently, and then the fluorescence indicator is liberated from the sensing unit (Figure 1). The bound and free fluorescent indicators have different properties, resulting in a signal change in proportion to the concentration of the analyte. The IDA concept was successfully applied for in vitro and in vivo enzymatic assays [16,17]. Herein we report a novel mechanism of tripartite IDA concept based on the transformation of inclusion complex to Exo complex of the indicator/receptor caused by the addition of analyte. The inclusion and Exo complex of the indicator has different fluorescent properties, resulting in a fluorescent change in proportion to the concentration of the analyte as proposed in Figure 1. In this study, a series of cyclodextrins and cucurbit[n]urils was screened for their ability to act as receptors, while a collection of environmental sensitive fluorescent dyes was evaluated for their binding affinity to the chosen receptor, in comparison with the deoxyadenosine. To the best of our knowledge, our study is the first successful case of using IDA for the detection of the bioactive deoxyadenosine and the quantitative analysis of cordycepin in commercially available *Ophipcordyceps* and *Cordyceps* products.

## 2. Results and Discussion

### 2.1. Selection of a Suitable Dye/Receptor Reporter Pair

**Figure 2:**
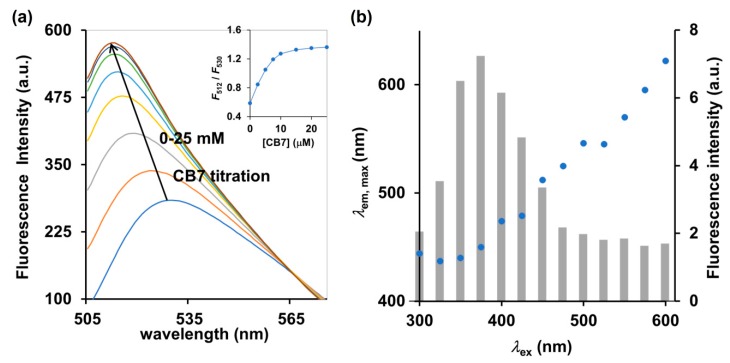
(**a**) Fluorescence spectrum and (inset) ratiometric change (*F*_512_/*F*_530_) of AOH^+^ (5 μM) upon addition of CB7 (0–25 μM) in 10 mM ammonium acetate buffer (pH 4.0), *λ*_ex_ = 498 nm. (**b**) Autofluorescence of *Cordyceps militaris* extracts at different excitation wavelengths (*λ*_ex_). The left and right y-axes represent the maximum emission wavelength (*λ*_em,max_, blue circle) and the corresponding fluorescence intensity (gray bar), respectively.

Pairwise combinations of candidate dyes and receptors were tested for their fluorescence properties compared to the uncomplexed counterparts. The receptor candidates comprise a variety of cyclodextrins and cucurbit[n]urils, and the candidates for fluorescent dyes included esculetin, cascade yellow, nonyl acridine orange, berberine (BE), 2-*p*-toluidinylnaphthalene-6-sulphonate (2,6-TNS) and acridine orange (AOH^+^) (see their chemical structures in Supplementary Materials Figure S1). We restricted the candidates to commercially available compounds, to ensure the cost-effectiveness and accessibility of our method. Among cyclodextrins and cucurbit[n]urils tested, cucurbit[7]uril (CB7) showed the highest increase in fluorescence response upon titration to the aqueous solution of the environment-sensitive fluorescent dye (Supplementary Materials Figure S1c). It should be noted that addition of CB7 to the AOH^+^ in 10 mM ammonium acetate at pH 4.0 caused an increase in fluorescence emission by approximately 100% as well as a hypochromic shift from 530 to 512 nm (Figure 2a). For free AOH^+^ under acidic condition, the ratio of *F*_512_ to *F*_530_ was 0.59 which then increased to 1.27 and 1.32 upon addition of two and three equivalents of CB7, respectively (Figure 2a inset), which indicated a strong affinity with a binding constant of 1.0 × 10^6^ M^−1^, as calculated by the least-square curve-fitting method. Shaikh et al. (2008), reported that the cationic AOH^+^ found under acidic condition (pH < 7) can strengthen the binding capacity between AOH^+^ and CB7 via ion–dipole interactions between the ureido carbonyl rims of CB7 and the charged AOH^+^ [18]. Other dyes such as BE and 2,6-TNS were also reported to exhibit significant increases in their fluorescent emissions upon binding to CB7 [19,20]. However, BE and 2,6-TNS require the excitation wavelengths of 343 and 328 nm, respectively, which may also induce strong autofluorescence from bright yellow *Cordyceps militaris* extracts and interfere with the detection. Indeed, we observed that excitation at 375 nm induced the highest intensity of autofluorescence of *Cordyceps militaris* centered at 450 nm (Figure 2b). The excitation beyond 475 nm yielded almost no autofluorescence as clearly shown in Figure 2b. Therefore, the AOH^+^/CB7 complex, which requires the excitation wavelength of 498 nm, was deemed a suitable IDA reporter pair for detection of cordycepin in *Cordyceps* and *Ophiocordyceps* products.

### 2.2. Fluorescence Detection of Deoxyadenosine and the Selectivity of IDA

**Figure 3:**
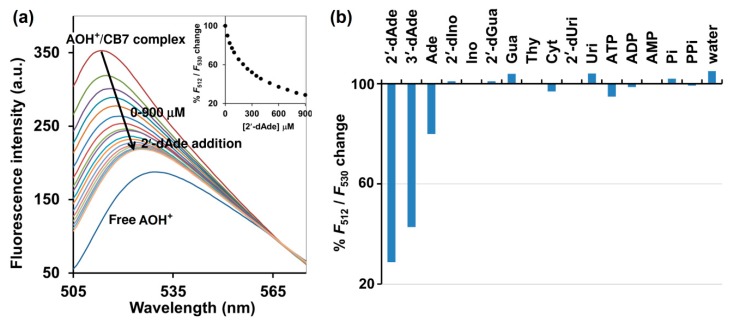
(**a**) Fluorescence spectrum of AOH^+^/CB7 complex upon addition of various concentrations of 2′-dAde. The percentages of change (*F*_512_/*F*_530_) are shown in the inset. (**b**) % change (*F*_512_/*F*_530_) of the AOH^+^/CB7 complex upon addition of various analytes at 900 μM. Measurement condition: 5 μM AOH^+^, 10 μM CB7, 10 mM ammonium acetate buffer (pH 4.0). *λ*_ex_ = 498 nm. Pi: inorganic phosphate; PPi: inorganic pyrophosphate.

A salient property of the CB7 macrocycle is its high affinity toward various molecules with *K*_a_ often in the range of 10^3^ M–10^9^ M^−1^, compared with cyclodextrin which rarely provides *K*_a_ values beyond higher than 10^3^ M^−1^ in aqueous media [19]. CB7 also shows the size and charge selectivity. Based on the inner cavity diameter and height of CB7 is 7.3 Å and 9.1 Å, respectively, the cavity volume is approximately 367 Å^3^ [20]. This strict size bestows high selectivity toward specific analytes that can fit in the cavity. Positively charged analytes showed generally higher affinities to CB7 than their non-charged counterparts by a factor of 10–100, due to their ability to form ion-dipole interactions with the carbonyl-fringed CB7 portals [20]. Isothermal titration calorimetry (ITC) suggested the 1:1 binding mode of CB7 toward AOH^+^, deoxyadenosines and adenosine. It was also revealed that CB7 bound 2′-dAde most strongly with the *K*_a_ of 5.62 × 10^4^ M^−1^, while 3- and 7-fold weaker binding was observed for 3′-dAde and Ade, respectively (Table 1 and Supplementary Materials Figure S2). The superior affinity of CB7 toward 2′-and 3′- dAde relative to Ade may be attributed to the lack of a hydroxyl group at the C2′ and C3′, respectively (Figure 1). Other deoxynucleosides, 2′-dIno and 2′-dGua, were also subjected to ITC with CB7, but no heat change was detected, which may be explained by the structural difference between 2′-dAde and these two analogs. In 2′-dAde, a primary amino group is located at C6 position, while in 2′-dIno this is replaced with a hydroxyl group. Although 2′-dGua has an NH_2_ substituent, it is located at C2 rather than C6 (Figure 1). Taken together, the results also suggest that the CB7 prefers binding with adenine over hypoxanthine, guanine and other pyrimidine bearing deoxynucleosides, leading to the preference toward 2′- and 3′-dAde over 2′-dIno, 2′-dGua and 2′-dUri (Figure 1). By considering the acid-base properties of adenosine and guanosine in aqueous solution, adenosine is in the monoprotonated form with three tautomers at N1, N7 and N3 [21], whereas guanosine exists in the neutral form with two tautomers at N1 and N7 [22]. The preference of CB7 toward deoxyadenosine can be ascribed to the high affinity of CB7 toward the positively charged monoprotonated adenine moiety in water, due to their ability to form ion-dipole interactions with the carbonyl-fringed CB7 portals [20]. The ITC also revealed that the binding affinity of CB7 toward AOH^+^ was 4.52 × 10^3^M^−1^ with hydrophobic interaction as a main driving force. The binding capability of AOH^+^/CB7 was 12.4- and 3.6-fold weaker than 2′-dAde/CB7 and 3′-dAde/CB7, respectively, suggesting that both 2′-dAde and 3′-dAde could displace AOH^+^ in the CB7 cavity. The fluorescence displacement assay was then employed using AOH^+^/CB7 as the reporter pair. Figure 3 showed that the fluorescence emission from the AOH^+^/CB7 complex, relative to the free AOH^+^ control, was reduced to 71% upon addition of 2′-dAde (0.9 mM) and red-shifted from 512 to 530 nm (Figure 3a). The percentage of reduction in *F*_512_/*F*_530_ was higher than 50% in the case of 2′-and 3′-dAde, but the value was diminished to 20% in the case of Ade. No significant change was observed when Ino, 2′-dIno, Gua, 2′-dGua, Uri, 2′-dUri, Cyt, Thy, ATP, ADP, AMP, inorganic phosphate (Pi), inorganic pyrophosphate (PPi) and purified water was added under the same condition. The fluorescent measurement strongly suggested that the AOH^+^/CB7 reporter pair is highly selective and suitable for the IDA-based detection of 3′-dAde, the bioactive compound in *Cordyceps* and *Ophicordyceps* products. Zhang et al. (2015) reported the spectroscopy and molecular modelling studies of the stable inclusion complex of 3′-dAde with α-cyclodextrins, and found electrostatic forces and hydrogen bonds to be the major contributors to their binding strength [23], which is in good agreement with what we observed in the dAde/CB7 complex in this study. To the best of our knowledge, our study is the first to investigate and report the high selectivity of CB7 toward both isomers of deoxyadenosine.

**Figure 4:**
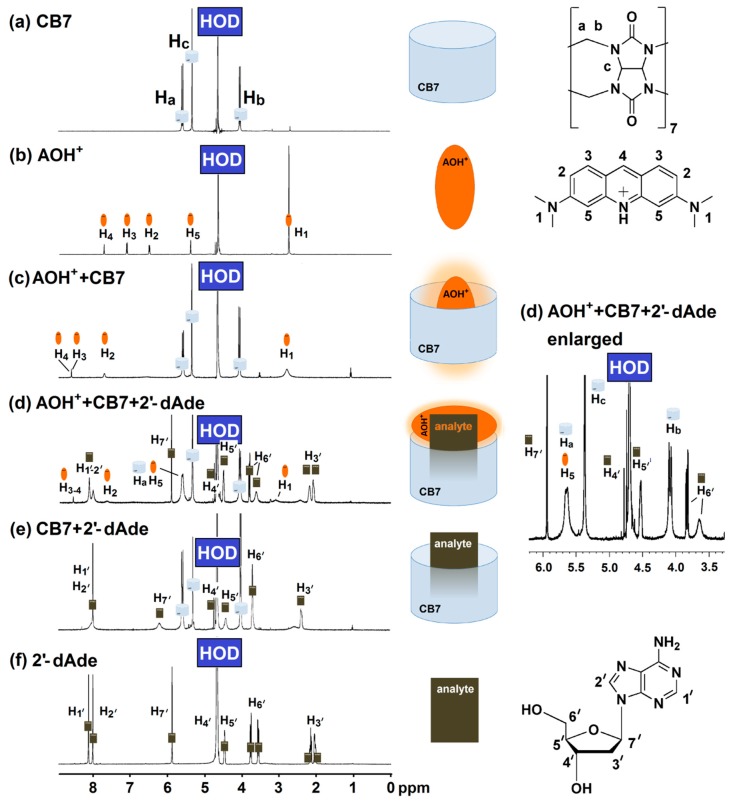
Proton nuclear magnetic resonance (400 MHz, D_2_O) spectrum of (**a**) CB7 (4 mM); (**b**) AO (2 mM); (**c**) mixture of AO (2 mM) and CB7 (4 mM); (**d**) mixture of AO (4 mM), CB7 (4 mM) and 2′-dAde (4 mM); (**e**) mixture of CB7 (4 mM) and 2′-dAde (4 mM); (**f**) 2′-dAde (20 mM).

The displacement of AOH^+^ by deoxyadenosine was further investigated using proton nuclear magnetic resonance (^1^H-NMR) and UV-Visible spectroscopy. ^1^H-NMR measurement provided an insight to the complexation between AOH^+^/CB7, 2′-dAde/CB7 and the mixture of AOH^+^/CB7/2′-dAde. The singlet peak at 2.8 ppm (H_1_) corresponds to the methyl group of the AOH^+^ that became broad after complexation with CB7 as shown in Figure 4b,c. The two protons at H3′ of 2′-dAde also merged and broadened, while the two singlet peaks of the adenine ring (H_1′_-H_2′_) collapsed into one singlet peak after complexation with CB7 (Figure 4e,f). However, the mixture of AOH^+^/CB7/2′-dAde yielded another unique spectrum which did not show any signs of free AOH^+^ (Figure 4d). Although further investigations in detail are required for structural elucidation of the host-guest interactions in the tripartite complex of AOH^+^/CB7/2′-dAde, it was clear that AOH^+^ was partially displaced, but not as a free molecule. The possibility of Exo complex formation between AOH^+^/CB7 and AOH^+^/sulfobutylether-β-cyclodextrin was suggested by Liu et al. (2009) and Sayed et al. (2017) [24,25]. In this study, the formation of Exo complex with 2′-dAde/CB7 may explain the fate of AOH^+^ after moved from the CB7 cavity (Figure 4d). They also reported that the Exo complex of AOH^+^/CB7 did not yield a strong fluorescence emission as observed in the case of inclusion complex [25], therefore transformation from an inclusion to Exo complex of AOH^+^/CB7 in the presence of deoxyadenosine enabling the unique fluorescent detection of deoxyadenosine using AOH^+^/CB7 reporter pair.

**Figure 5:**
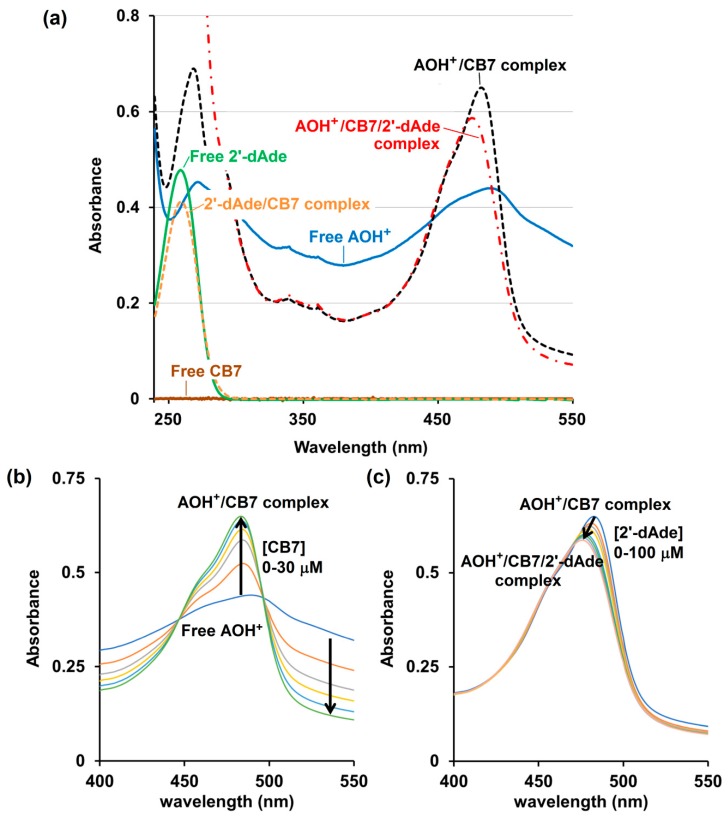
UV-Visible spectrum (JASCO V-530, 10 mM ammonium acetate buffer, pH 4.0) of (**a**) free CB7 (10 μM, solid brown line), free protonated acridine orange (AOH^+^, 10 μM, solid blue line), free 2′-deoxyadenosine (2′-dAde, 30 μM, solid green line), 2′-dAde/CB7 complex (30/30 μM, respectively, orange dash line), AOH^+^/CB7 complex (10/30 μM, respectively, black dash line) and AOH^+^/CB7/2′-dAde complex (10/30/100 μM, respectively, red dash dot line); (**b**) AOH^+^(10 μM) after being titrated with CB7 and (**c**) addition of 2′-dAde to the AOH^+^/CB7 complex (10/30 μM, respectively).

UV-Visible spectroscopy analysis confirms that the free CB7, 2′-dAde and the CB7/2′-dAde complex do not have any absorbance beyond 300 nm, while the free AOH^+^ displays a strong absorbance centered at 498 nm (Figure 5a). The complex formation between the AOH^+^ and CB7 increased the absorbance at 483 nm sharply with the two isosbestic points at 452 and 502 nm upon addition of CB7 to the AOH^+^ in 10 mM ammonium acetate buffer solution (pH 4.0) (Figure 5b). However, the addition of 2′-dAde to AOH^+^/CB7 did not reverse the UV-Vis spectrum of AOH^+^/CB7 to the free AOH^+^, but only the slight intensity reduction and blue shift of the *λ*_max_ from 483 to 476 nm after addition of 2′-dAde were observed (Figure 5c). This result agrees well with the ^1^H-NMR spectrum that 2′-dAde could not liberate the AO from the CB7 cavity, but instead spurred the formation of a three-component AOH^+^/CB7/2′-dAde complex.

For comparison, the ^1^H-NMR spectrum of 2′-dIno and CB7 was obtained and shown in Supplementary Materials Figure S3. The ^1^H-NMR spectrum of 2′-dIno and CB7 demonstrated that both 2′-dIno and CB7 existed in the free forms, without any interaction with each other. The ^1^H-NMR result also corroborated the ITC and fluorescence measurements that CB7 exhibited a strong binding affinity toward deoxyadenosine over other deoxynucleosides, nucleosides and nucleotides.

We concluded that the detection of deoxyadenosine using AOH^+^/CB7 reporter pair based on the tripartite IDA principle demonstrated a high selectivity, thus suitable for the determination of the bioactive deoxyadenosine in *Cordyceps* and *Ophiocordyceps* products.

### 2.3. Fluorescence Detection of Deoxyadenosine in Commercially Available Cordyceps and Ophiocordyceps Products Based on Tripartite IDA

**Figure 6:**
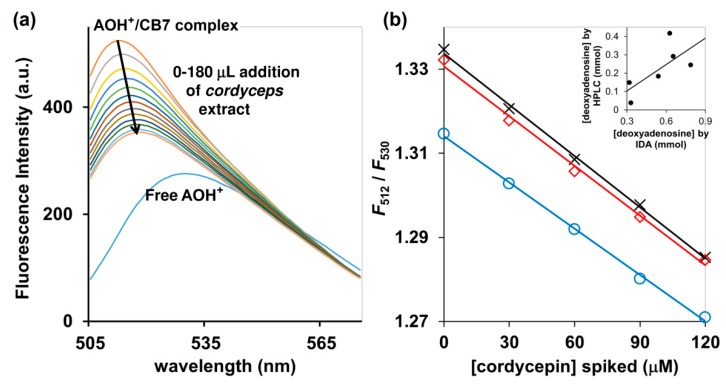
(**a**) Fluorescence detection of deoxynucleosides in *Ophiocordyceps* and *Cordyceps* commercial products using AOH^+^/CB7 as a reporter, performed in 10 mM ammonium acetate buffer (pH 4.0); (**b**) *F*_512_/*F*_530_ of AOH^+^/CB7 complex upon addition of *Ophiocordyceps* and *Cordyceps* product number 1 (blue circle), number 2 (red square) and number 3 (black cross), all of which had been spiked with cordycepin; (inset) relative concentrations of deoxynucleotides determined by HPLC and tripartite IDA proposed in this study.

Addition of an aqueous extract of the commercially available *Cordyceps* and *Ohiocordyceps* products to the AOH^+^/CB7 inclusion complex resulted in fluorescent quenching accompanied by the red shift from 512 to 530 nm (Figure 6a). This suggests that cordycepin present in the sample could switch the AOH^+^ molecule from an inclusion complex to the Exo complex, as also demonstrated by ^1^H-NMR and UV-Vis spectroscopy. The fluorescence change (*F*_512_/*F*_530_) was linearly correlated with the amount of cordycepin spiked, with the R^2^ > 0.99 (Figure 6b). The concentrations of cordycepin determined by HPLC were slightly lower than the values obtained from our IDA method, but both still exhibited some agreement despite the large variety of ingredients in the commercially available *Cordyceps* and *Ophiocordyceps* products (Figure 6b inset). Taken together, the bioactive cordycepin with several reported health benefits can be evaluated in commercial *Cordyceps* and *Ohiocordyceps* products using tripartite IDA with AOH^+^/CB7 as a reporter pair in 10 mM ammonium acetate buffer (pH 4.0) as an approximate analysis. Besides its rapidity, the newly developed IDA method is also environmentally-friendly because it produced less chemical wastes—the volume of each assay is limited to only 3 mL, in contrast to conventional HPLC techniques which demand large amounts of mobile phase for column conditioning, sample elution, and post-assay column cleaning [26]. The method will be highly suitable for quality control of the *Cordyceps* and *Ohiocordyceps* commercial products, especially in the production line.

In summary, we reported the highly selective deoxyadenosine detection based on IDA, using AOH^+^/CB7 as a reporter. Addition of cordycepin to the highly fluorescent AOH^+^/CB7 complex resulted in a unique tripartite AOH^+^/CB7/dAde complex with diminished fluorescence, and such reduction in emission intensity serves as the basis for our novel sensing system. Although the developed tripartite IDA system is already quite specific to deoxyadenosine, our research group is currently working toward achieving better differentiation between the 2′-dAde and 3′-dAde, and between the bioactive cordycepin and adenosine, in order to improve the accuracy of cordycepin quantification in the commercial *Cordyceps* and *Ophiocordyceps* products even further.

## 3. Materials and Methods

### 3.1. Chemicals

Chemicals and reagents used in this study were of analytical grade or higher. Ammonia solution, glacial acetic acid, and HPLC grade acetonitrile were purchased from Merck, Darmstadt, Germany. Sodium dihydrogen orthophosphate and sodium hydroxide were obtained from Ajax Finechem Pty Ltd., Auckland, New Zealand. Cyclodextrins, cordycepin, 2′-deoxyguanosine, uridine, and 2′-deoxyadenosine were commercially available from Wako Pure Chemical Industry, Ltd., Osaka Japan. Sodium pyrophosphate decahydrate, adenosine 5′-triphosphate disodium salt (ATP), adenosine 3′-monophosphate (AMP), adenosine 5′-diphosphate disodium (ADP), adenosine, and cucurbit[n]urils were obtained from Sigma-Aldrich Co. St. Louis, MO, USA. Cytidine, 2′-deoxyuridine, inosine, thymidine, and 2′-deoxyinosine were purchased from FUJIFILM Wako Pure Chemical Corporation, Japan. Guanosine was obtained from Alfa Aesar, Thermo Fisher Scientific, Heysham, UK. Acridine orange (AO) and berberine (BE) was purchased from Invitrogen Company, Eugene, Oregon, OR USA. Deionized water was prepared using a Millipore Milli Q-Plus system (Millipore, Bedford, MA, USA). Deuterium oxide (D_2_O) was purchased from Cambridge Isotope Laboratories, Inc., Tewksbury, MA, USA.

### 3.2. Spectroscopy

To a solution of 10 mM ammonium acetate buffer pH 4.0 (3 mL), the stock solution of the fluorescent dye was added and then the fluorescence emission was measured by a spectrofluorometer (JASCO FP6500, Tokyo, Japan). A receptor was serially titrated to the dye solution until the saturation of fluorescence emission was observed. The procedure was repeated by varying the receptors and dyes. The autofluorescence of the *C. millitaris* extract, which appears as a bright yellow color, was evaluated by measuring the fluorescence emission spectrum of the extract at the excitation wavelength between 300–600 nm.

A stock solution of deoxyadenosine and other deoxynucleoside and nucleoside was added serially to the solution of 10 mM ammonium acetate buffer pH 4.0 (3 mL) containing 5 μM acridine orange and 10 μM CB7. The fluorescence spectrum was then recorded by a spectrofluorometer (JASCO FP6500, Tokyo, Japan). The analysis of *Ophiocordyceps* and *Cordyceps* samples was conducted under similar condition. UV-Visible spectrum was recorded using a JASCO V-530 (Tokyo, Japan). ^1^H-NMR spectrum was acquired on Bruker Ascend^TM^ 400 (Bruker BioSpin, Billerica, Massachusetts, MA USA).

### 3.3. Isothermal Titration Calorimetric (ITC) Analysis

Acridine orange, cordycepin, adenosine, 2′-deoxyadenosine and 2′-deoxyinosine were set as titrands whereas the CB7 solution was used as a titrant. The measurement was performed in Microcal PEAQ-ITC (Malvern Instrument, Malvern, UK). The sample cell was loaded with 0.5 mM CB7, while the reference cell was filled with 10 mM ammonium acetate buffer solution pH 4.0. The syringe was filled with 5 mM titrand solution. Microcal PEAQ-ITC Analysis Software was used to analyze calorimetric data for elucidation of binding mechanism and the value of association constant.

### 3.4. Samples Preparation, Extraction and HPLC Analysis

Commercially available *Cordyceps* and *Ohiocordyceps* products were dried at 60 °C for 24 h. All samples were ground into powder (approximately 50 meshes). Then 1.0 g of sample was weighed and added to 20 mL of deionized water. The sample mixture was placed into an ultrasonic bath for 2 h. After extraction, the extract was cooled down to the room temperature, then centrifuged at 18,225× *g* (WiseSpin CF-10, Daihan Scientific, Gangwon, Korea) for 5 min to obtain the clear supernatant. The supernatant was filtered through 0.45 μm Supor^®^ Membrane, Acrodisc^®^ syringe filters before analysis. The HPLC analysis was conducted according to Ip et al. (1985) with slight modifications [27]. HPLC analysis of all samples was carried out using a Waters 600 HPLC system equipped with a C-18 reverse phase ODS column (Zorbax ODS, 4.6 mm × 250 mm, 5μm). Isocratic elution was carried out by using 0.1 M triethyl ammonium acetate (TEAA) pH 7.1/acetonitrile (98/2 *v*/*v*) as the mobile phase at a flow rate of 1.0 mL/min. Sample injection volume was 10 μL. The detection was done at 254 nm using a photo-diode array detector (Waters 996).

## Figures and Tables

**Table 1:** Thermodynamic parameters of complex formation between CB7 and guests (AOH^+^ and different nucleosides) assessed by isothermal titration calorimeter in 10 mM ammonium acetate buffer (pH 4.0).

Guest	*K*_a_ (M^−1^)	ΔH (kcal/mol)	ΔG (kcal/mol)	-TΔS (kcal/mol)	Major Interaction
AOH^+^	4.52 × 10^3^	15.8 ± 5.1	−4.99	−20.8	Hydrophobic
2′-dAde	5.62 × 10^4^	−10.3 ± 0.2	−6.48	3.79	Hydrogen bonding
3′-dAde	1.64 × 10^4^	−12.0 ± 0.4	−5.75	6.21	Hydrogen bonding
Ade	8.06 × 10^3^	−8.6 ± 0.3	−5.33	3.23	Hydrogen bonding
2′-dIno 2′-dGua	No binding observed				

## Supplementary Materials

The following are available online at https://www.mdpi.com/1420-3049/25/9/2045/s1, Figure S1: Chemical structures of macrocyclic receptors (**a**) and fluorescent dyes (**b**) tested in this study; (**c**) Fluorescent response of the dye without (*F*_0_) and with 5 equivalent molar of β-CD (white) and CB7 (gray) in 10 mM ammonium acetate buffer (pH 5.0); Figure S2: ITC analysis (MicroCal PEAQ-ITC, Malvern Instrument, UK) of CB7 (0.5 mM) and (**a**) AOH^+^ (4 mM), (**b**) 2′-deoxyadenosine (5 mM) and (**c**) 3′-deoxyadenosine (5 mM) in 10 mM ammonium acetate buffer (pH 4.0); Figure S3: ^1^H-NMR (Bruker Ascend^TM^ 400, D_2_O) of the (**a**) CB7 (4 mM), (**b**) 2′-deoxyinosine (4 mM) and (**c**) the mixture between 2′-deoxyinosine and CB7 (4 mM each).

## References

[1] Su N.W., Wu S.H., Chi C.W., Liu C.J., Tsai T.H., Chen Y.J. (2017). Metronomic cordycepin therapy prolongs survival of oral cancer-bearing mice and inhibits epithelial-mesenchymal transition. Molecules.

[2] Wang Z., Wu X., Liang Y.N., Wang L., Song Z.X., Liu J.L., Tang Z.S. (2016). Cordycepin induces apoptosis and inhibits proliferation of human lung cancer cell line H1975 via inhibiting the phosphorylation of EGFR. Molecules.

[3] Wang X.A., Xiang S.S., Li H.F., Wu X.S., Li M.L., Shu Y.J., Zhang F., Cao Y., Ye Y.Y., Bao R.F. (2014). Cordycepin induces S phase arrest and apoptosis in human gallbladder cancer cells. Molecules.

[4] Zhao J., Xie J., Wang L.Y., Li S.P. (2014). Advanced development in chemical analysis of *Cordyceps*. J. Pharm. Biomed. Anal..

[5] Phan C.W., Wang J.K., Cheah S.C., Naidu M., David P., Sabaratnam V. (2018). A review on the nucleic acid constituents in mushrooms: Nucleobases, nucleosides and nucleotides. Crit. Rev. Biotechnol..

[6] Qin P., Li X., Yang H., Wang Z.Y., Lu D. (2019). Therapeutic potential and biological applications of cordycepin and metabolic mechanisms in cordycepin-producing fungi. Molecules.

[7] Wong Y.Y., Moon A., Duffin R., Barthet-Barateig A., Meijer H.A., Clemens M.J., de Moor C.H. (2010). Cordycepin inhibits protein synthesis and cell adhesion through effects on signal transduction. J. Biol. Chem..

[8] Cunningham K.G., Manson W., Spring F.S., Hutchinson S.A. (1950). Cordycepin, a metabolic product isolated from cultures of *Cordyceps militaris* (Linn.) Link. Nature.

[9] Rao Y.K., Chou C.H., Tzeng Y.M. (2006). A simple and rapid method for identification and determination of cordycepin in *Cordyceps militaris* by capillary electrophoresis. Anal. Chim. Acta.

[10] Wang Z., Li N., Wang Y., Du L., Ji X., Yu A., Zhang H., Qiu F. (2013). Simultaneous determination of nucleosides and their bases in *Cordyceps sinensis* and its substitutes by matrix solid-phase dispersion extraction and HPLC. J. Sep. Sci..

[11] Guo F.Q., Li A., Huang L.F., Liang Y.Z., Chen B.M. (2006). Identification and determination of nucleosides in *Cordyceps sinensis* and its substitutes by high performance liquid chromatography with mass spectrometric detection. J. Pharm. Biomed. Anal..

[12] Hu H., Xiao L., Zheng B., Wei X., Ellis A., Liu Y.M. (2015). Identification of chemical markers in *Cordyceps sinensis* by HPLC-MS/MS. Anal. Bioanal. Chem..

[13] Xie J.W., Huang L.F., Hu W., He Y.B., Wong K.P. (2010). Analysis of the main nucleosides in *Cordyceps sinensis* by LC/ESI-MS. Molecules.

[14] Zhao H.Q., Wang X., Li H.M., Yang B., Yang H.J., Huang L. (2013). Characterization of nucleosides and nucleobases in natural *Cordyceps* by HILIC-ESI/TOF/MS and HILIC-ESI/MS. Molecules.

[15] You L., Zha D., Anslyn E.V. (2015). Recent Advances in Supramolecular Analytical Chemistry Using Optical Sensing. Chem. Rev..

[16] Hennig A., Bakirci H., Nau W.M. (2007). Label-free continuous enzyme assays with macrocycle-fluorescent dye complexes. Nat. Methods.

[17] Norouzy A., Azizi Z., Nau W.M. (2015). Indicator displacement assays inside live cells. Angew. Chem. Int. Ed..

[18] Shaikh M., Mohanty J., Singh P.K., Nau W.M., Pal H. (2008). Complexation of acridine orange by cucurbit[7]uril and β-cyclodextrin: Photophysical effects and pK a shifts. Photochem. Photobiol. Sci..

[19] Dsouza R.N., Pischel U., Nau W.M. (2011). Fluorescent dyes and their supramolecular receptor/guest complexes with macrocycles in aqueous solution. Chem. Rev..

[20] Sinn S., Biedermann F. (2018). Chemical sensors based on cucurbit[n]uril macrocycles. Isr. J. Chem..

[21] Kapinos L.E., Operschall B.T., Larsen E., Sigel H. (2011). Understanding the acid-base properties of adenosine: The intrinsic basicities of N1, N3 and N7. Chem. Eur. J..

[22] Jang Y.H., Goddard W.A., Noyes K.T., Sowers L.C., Hwang S., Chung D.S. (2003). p*K*_a_ values of guanine in water: Density functional theory calculations combined with Poisson-Boltzmann Continuum-Solvation model. J. Phys. Chem. B.

[23] Zhang J.Q., Wu D., Jiang K.M., Zhanga D., Zheng X., Wan C.P., Zhu H.Y., Xie X.G., Jin Y., Lin J. (2015). Preparation, spectroscopy and molecular modeling studies of the inclusion complex of cordycepin with cyclodextrins. Carbohydr. Res..

[24] Liu J., Jiang N., Ma J., Du X. (2009). Insight into unusual downfield NMR shifts in the inclusion complex of acridine orange with cucurbit[7]uril. Eur. J. Org. Chem..

[25] Sayed M., Jha S., Pal H. (2017). Complexation induced aggregation and deaggregation of acridine orange with sulfobutylether-β-cyclodextrin. Phys. Chem. Chem. Phys..

[26] Christian G.D. (1994). Analytical Chemistry.

[27] Ip C.Y., Ha D., Morris P.W., Puttemans M.L., Venton D.L. (1985). Separation of nucleosides and nucleotides by reversed-phase high-performance liquid chromatography with volatile buffers allowing sample recovery. Anal. Biochem..

