# Supplementary material for: Fluorescence Detection of Deoxyadenosine in Cordyceps spp. by Indicator Displacement Assay
Source: Molecules. 2020 Apr 28;25(9):2045. doi: 10.3390/molecules25092045 (PMC7248813; doi:10.3390/molecules25092045)
Supplement: Supplementary file 1 [file molecules-25-02045-s001.pdf]

Supplementary Materials

Fluorescence Detection of deoxyadenosine in *Cordyceps* spp. by Indicator Displacement Assay

Arinta Agnie Dewantari [1], Nattha Yongwattana [1], Panwajee Payongsri [1], Sawinee Seemakhan [2], Suparerk Borwornpinyo [1,2], Akio Ojida [3] and Jirarut Wongkongkatep [1,*]

[1] Department of Biotechnology, Faculty of Science, Mahidol University, Bangkok 10400, Thailand
[2] Excellent Center for Drug Discovery, Faculty of Science, Mahidol University, Bangkok 10400, Thailand
[3] Graduate School of Pharmaceutical Sciences, Kyushu University, 3-1-1 Maidashi, Higashi-ku, Fukuoka 812-8582, Japan
* Correspondence: jirarut.chu@mahidol.ac.th

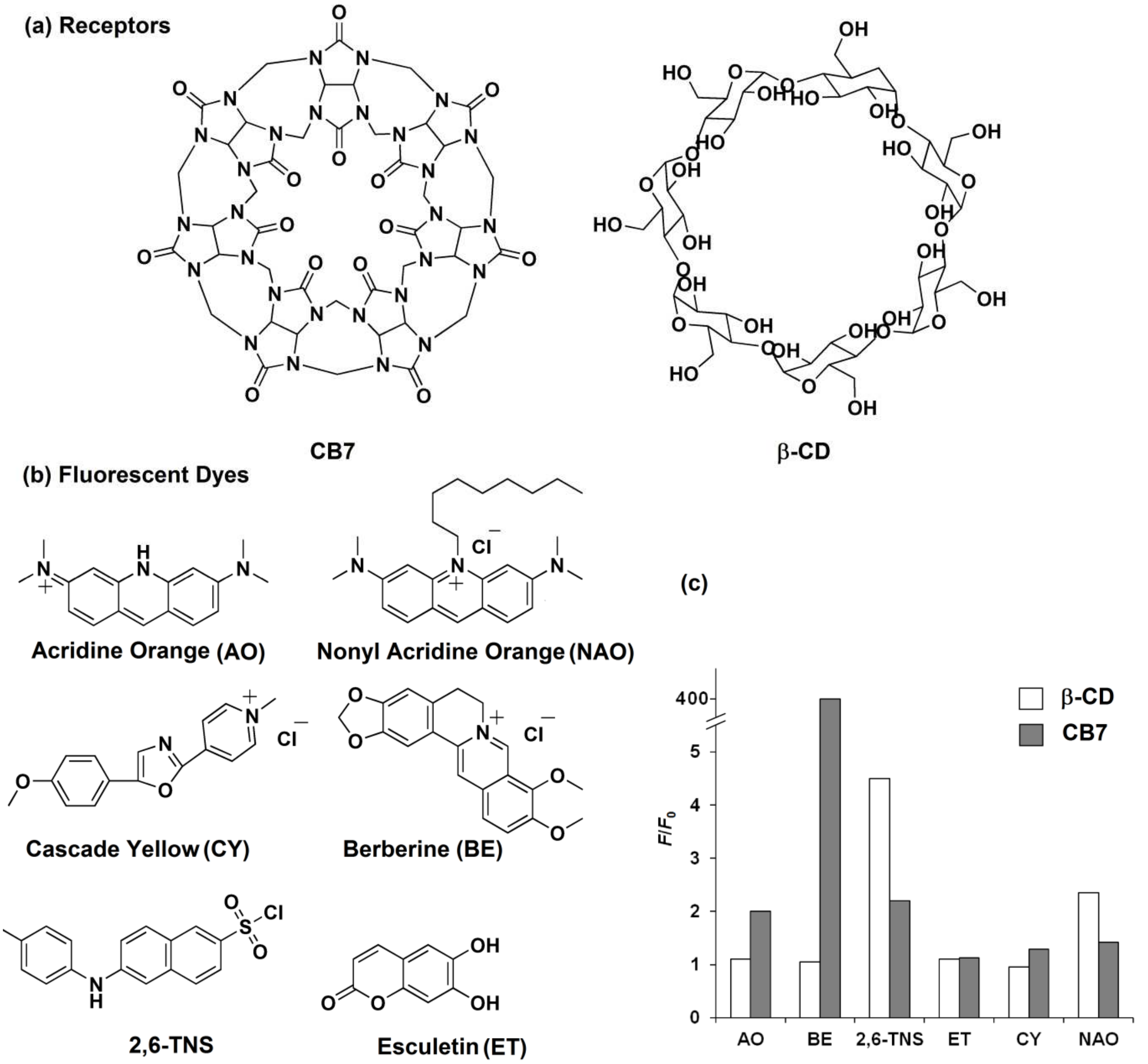

Figure S1. Chemical structures of macrocyclic receptors (**a**) and fluorescent dyes (**b**) tested in this study; (**c**) Fluorescent response of the dye without (*F_0*) and with 5 equivalent molar of β-CD (white) and CB7 (gray) in 10 mM ammonium acetate buffer (pH 5.0)

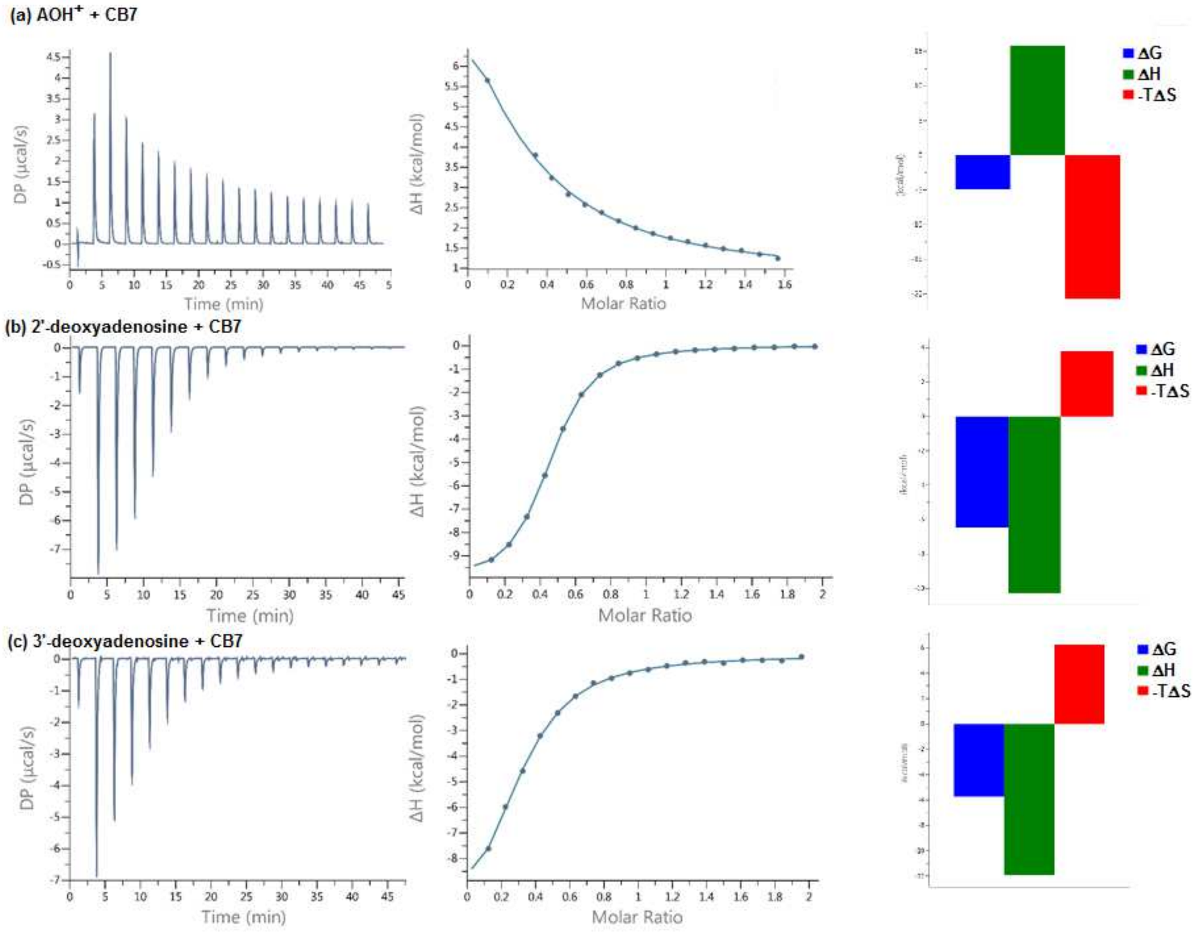

Figure S2. ITC analysis (MicroCal PEAQ-ITC, Malvern Instrument, UK) of CB7 (0.5 mM) and (**a**) AOH^+ (4 mM), (**b**) 2′-deoxyadenosine (5 mM) and (**c**) 3′-deoxyadenosine (5 mM) in 10 mM ammonium acetate buffer (pH 4.0). Blue : ΔG, Green : ΔH, Red : -TΔS (kcal/mol).

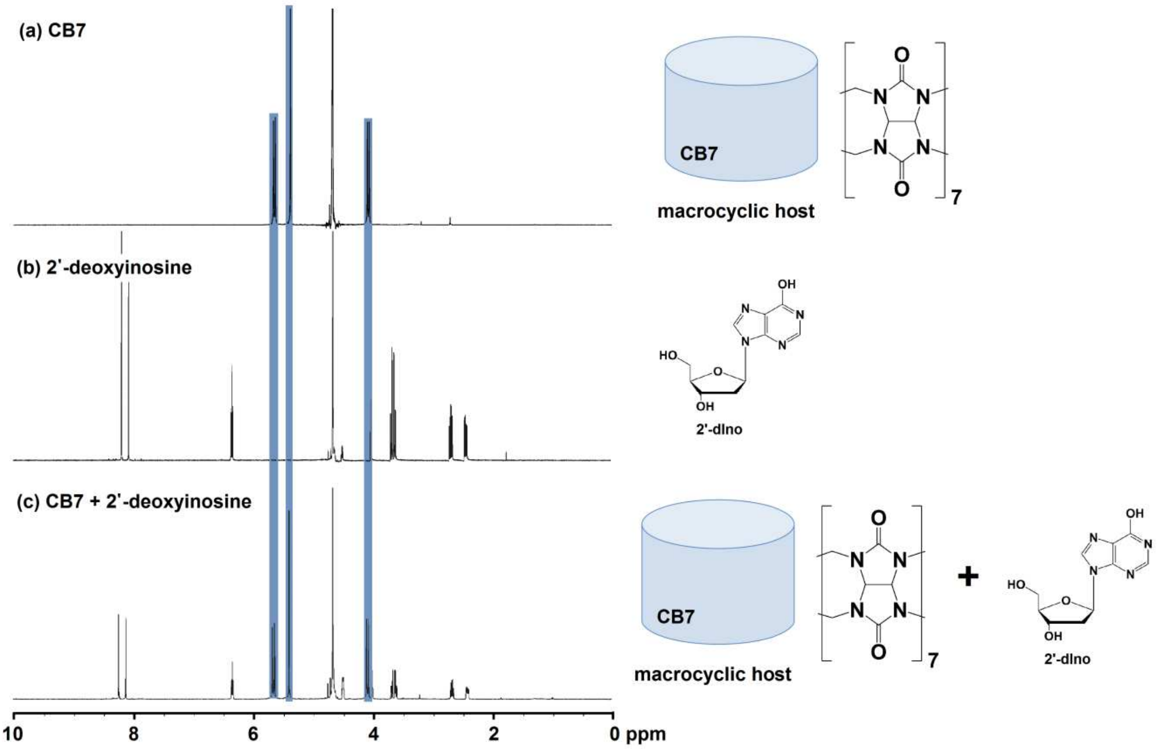

Figure S3. ^{1}HNMR (Bruker Ascend™ 400, D_2O) of the (**a**) CB7 (4 mM), (**b**) 2′-deoxyinosine (4 mM) and (**c**) the mixture between 2′-deoxyinosine and CB7 (4 mM each). Peaks of the CB7 were highlighted in blue.
